# Supplementary material for: Rapid high-throughput isolation and purification of chicken myoblasts based on deterministic lateral displacement microfluidic chips
Source: PLoS One. 2024 Dec 5;19(12):e0301309. doi: 10.1371/journal.pone.0301309 (PMC11620410; doi:10.1371/journal.pone.0301309)
Supplement: S1 Appendix — (DOCX) [file pone.0301309.s001.docx]

Supplementary information

S1. tissue slice

S1.1 Experimental equipment

Supplementary Table 1

| **Name** | **Manufacturer** | **Model** |
| --- | --- | --- |
| Dehydrator | DIAPATH | Donatello |
| Embedding Machine | Wuhan Junjie Electronics Co., Ltd. | JB-P5 |
| Pathology Microtome | Leica Instruments Co., Ltd., Shanghai | RM2016 |
| Freezing Stage | Wuhan Junjie Electronics Co., Ltd. | JB-L5 |
| Tissue Flotation Workstation | Zhejiang Jinhua Kedi Instrument Equipment Co., Ltd. | KD-P |
| Oven | Tianjin Laibori Instrument Equipment Co., Ltd. | GFL-230 |
| Cryostat | Thermo Fisher Scientific (China) Co., Ltd. | CRYOSTAR NX50 |
| Adhesive Slides (Paraffin Sections) | Servicebio | G6012-1 (White Paint Ribbon) |
| Adhesive Slides (Frozen Sections) | Servicebio | G6012-2 (White Paint Ribbon) |
| Cover Slips | Jiangsu Shitai Laboratory Equipment Co., Ltd. | 10212432C |
| Upright Optical Microscope | Nikon, Japan | Nikon-Eclipse-E100 |
| Imaging System | Nikon, Japan | Nikon DS-U3e |

Experimental reagents

Supplementary table 2

| **Reagent Name** | **Manufacturer** | **Catalog Number** |
| --- | --- | --- |
| Anhydrous Ethanol | China National Pharmaceutical Group Chemical Reagent Co., Ltd. | 100092683 |
| Xylene | China National Pharmaceutical Group Chemical Reagent Co., Ltd. | 10023418 |
| n-Butanol | China National Pharmaceutical Group Chemical Reagent Co., Ltd. | 100052190 |
| Eco-Friendly Deparaffinizing Solution | Servicebio | G1128 |
| Universal Tissue Fixative | Servicebio | G1101 |
| Hematoxylin-Eosin (H&E) Staining Kit | Servicebio | G1076 |
| Neutral Gum | China National Pharmaceutical Group Chemical Reagent Co., Ltd. | 10004160 |

S1.2 Tissue sections

Tissue sections were prepared according to the SOP (Standard Operating Procedure) for pathology tissue collection, fixation, embedding, paraffin sectioning, and frozen sectioning as per Servicebio's guidelines:

**Tissue Collection:** Follow standard procedures for tissue collection ensuring proper handling and preservation.

**Tissue Fixation:** Use Servicebio's Universal Tissue Fixative (Catalog: G1101) according to the recommended fixation protocol for the specific tissue types.

**Embedding:** Embed the fixed tissues in paraffin wax using the embedding machine (e.g., JB-P5 from Wuhan Junjie Electronics Co., Ltd.).

**Paraffin Sectioning:** Cut paraffin sections using a microtome (e.g., RM2016 from Leica Instruments Co., Ltd., Shanghai).

**Frozen Sectioning:** For frozen sections, use Servicebio's Eco-Friendly Deparaffinizing Solution (Catalog: G1128) to prepare tissues and then cut the sections using a cryostat (e.g., CRYOSTAR NX50 from Thermo Fisher Scientific (China) Co., Ltd.).

**Staining:** Perform staining procedures such as Hematoxylin-Eosin (H&E) staining using Servicebio's H&E Staining Kit (Catalog: G1076) to visualize tissue structures.

Paraffin section processing: After rinsing six fixed embryonic stages of chest muscle samples with tap water, the chest muscle tissue was dehydrated twice with a series of alcohol concentrations. The dehydration process was as follows: 75% alcohol for 4 hours, 85% alcohol for 4 hours, overnight in 95% alcohol, and 100% alcohol for 2 hours. After dehydration, the tissue specimens were treated with xylene three times, embedded in paraffin, trimmed, and cut into 4 μm-thick sections. The paraffin sections were placed in a 40°C water bath for approximately 5 minutes, transferred onto slides, air-dried for 30 minutes, baked overnight at 45°C, deparaffinized with two different concentrations of xylene for 10 minutes each time, dehydrated twice with 100% ethanol for 3 minutes each, dehydrated with 95% and 80% ethanol for 1 minute each, and finally rinsed with distilled water for 5 minutes. The sections were stained with Hematoxylin and Eosin (H&E).

S1.3 Staining

Here's a structured translation of the process for preparing tissue sections:

**Paraffin Section Dewaxing to Water:**

Immerse the sections sequentially in Eco-Friendly Deparaffinizing Solution I for 20 minutes, followed by Eco-Friendly Deparaffinizing Solution II for 20 minutes.

Transfer to Anhydrous Ethanol I for 5 minutes, then Anhydrous Ethanol II for 5 minutes.

Submerge in 75% ethanol for 5 minutes, followed by rinsing with tap water.

**Thawed Section Re-fixation:**

Recover frozen sections from -20°C freezer to room temperature.

Fix in Tissue Fixative Solution for 15 minutes, then rinse with running water.

**Pre-treatment:**

Treat sections in High-Definition Permanent Stain Pre-treatment Solution for 1 minute.

**Hematoxylin Staining:**

Immerse sections in Hematoxylin Stain Solution for 3-5 minutes.

Rinse in tap water, then differentiate in Differentiation Solution.

Rinse again in tap water, followed by blueing in Bluing Solution.

Rinse thoroughly with running water.

**Eosin Staining:**

Dehydrate sections in 95% ethanol for 1 minute, then immerse in Eosin Stain Solution for 15 seconds.

**Dehydration and Mounting:**

Dehydrate the sections sequentially in Anhydrous Ethanol I for 2 minutes, Anhydrous Ethanol II for 2 minutes, Anhydrous Ethanol III for 2 minutes, n-Butanol I for 2 minutes, n-Butanol II for 2 minutes, Xylene I for 2 minutes, and Xylene II for 2 minutes to achieve transparency.

Mount with Neutral Gum.

**Microscope Examination, Image Acquisition, and Analysis:**

Examine the slides under a microscope.

Capture and analyze images.

S2. Cell suspension solution preparation

The experimental animal samples chosen were 11-day-old Wen Chang chicken eggs from Hainan. Cell suspensions were prepared using collagenase digestion method.

Key reagents used in the experiment include: Type I collagenase purchased from Solarbio, trypsin purchased from Sigma, DMEM low glucose medium purchased from Sigma, fetal bovine serum purchased from Biyun Tian, Pax7 antibody purchased from Santa Cruz, FITC-conjugated goat anti-mouse IgG purchased from Biyun Tian, PBS solution purchased from Gibco, double antibiotics mixture (penicillin, streptomycin) purchased from Gibco, 4% paraformaldehyde (100 ml) purchased from Biosharp, PBST buffer (500 ml) purchased from Biyun Tian, 0.5% Triton-X100 (100 ml) purchased from Biyun Tian, and 1% BSA (100 ml) purchased from Biosharp.

After disinfecting 11-day-old chicken embryos, the pectoral muscles were isolated in PBS dishes containing double antibiotics. The muscles were washed once with PBS containing double antibiotics, minced in a PBS dish, and transferred to centrifuge tubes. After washing with DMEM, the supernatant was discarded, and an appropriate amount of DMEM was added to the minced muscle in the centrifuge tube. Trypsin digestion was performed at 37°C for 1 hour until cells were completely dispersed. Digestion was terminated using DMEM containing 10% FBS. The dispersed cells were sequentially filtered through 200 and 400 mesh cell sieves. The filtrate was centrifuged at 1000 rpm for 10 minutes, supernatant discarded, and cells resuspended in DMEM containing 10% FBS. After cell counting, the cell concentration was adjusted to 1×10^6 cells/mL.

S3. Chip processing, manufacturing

Circular micropost diameter D = 50 μm, circular micropost spacing G = 20 μm, deflection angle α = 5.7°, lateral spacing λ = longitudinal spacing = 65.3 μm. (Figure S1)

After determining various parameters for the DLD chip, the chip is processed and fabricated according to the following steps.

Chip fabrication mainly involves silicon wafer pretreatment, SU-8 resist coating, pre-baking, photolithography, exposure, mid-baking, post-baking, modification, molding, demolding, punching, and bonding. A process flowchart for PBMS chip fabrication, include silicon wafer cleaning and drying, uniform SU-8 resist coating on a single-crystal silicon surface, exposure under a lithography machine, multiple development and drying cycles, pouring PDMS into a mold, removing after PDMS solidification through heating, and bonding with a glass substrate to form the chip.


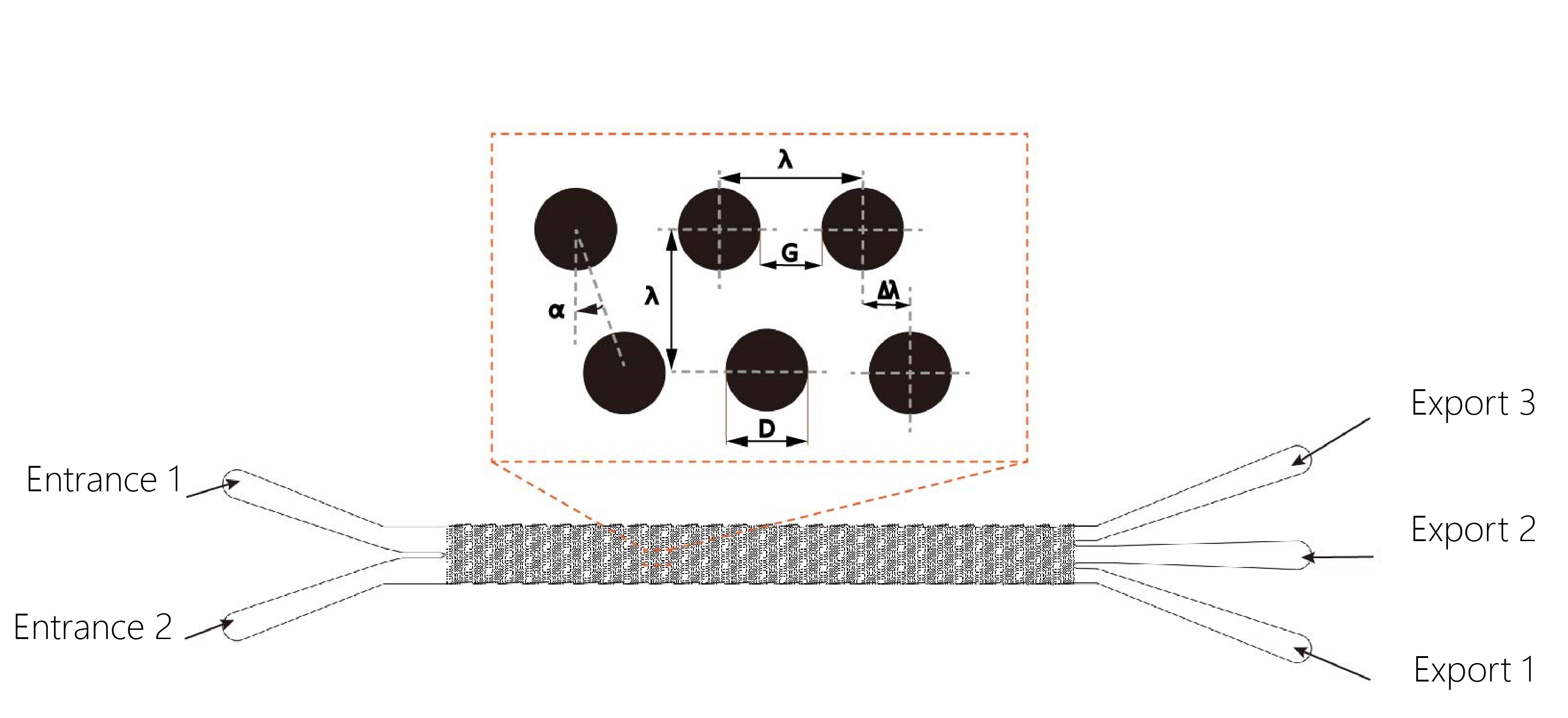


Figure S1 Designed for the chip structure and parameters

S4. Deterministic lateral displacement (DLD) experiment

Experimental Instruments

Inverted microscope (OLYMPUS IX73)，High-speed camera (CCD)，Dual-channel high-precision syringe pump (Cchippump01-BD, Zhongxin Qiheng)，Desktop computer，Experimental Steps

**Preparation:** Before passing the cell suspension through the DLD chip, perform pre-treatment on the chip. First, pass deionized water through the chip to increase the hydrophilicity of the channels. Then, pass PBS solution through the chip to prevent any residual liquid from harming the cells.

**Setup:** Place the treated chip on the microscope stage.

Connect the syringe pump and the chip inlet using tubing. Introduce sheath fluid and cell suspension into the inlets. The outlet of the chip is connected to a collection cup using tubing. The flow rates at the inlets are controlled by two independent syringe pumps.

**Operation:** At the appropriate flow rates, "zigzag" and collision trajectories will appear in the middle of the channel.

Use a hemocytometer and cell counter to count the cells at the inlet and outlet. Calculate the recovery rate and purity of the cells at the outlet.

**Calculations:** Recovery Rate: The ratio of the number of target cells collected at a specific outlet to the total number of target cells collected at all outlets.

Purity: The ratio of the number of target cells collected at the target outlet to the total number of cells collected at the same outlet.

**Analysis:** Analyze the shape and size of the cells at different outlets and statistically determine the separation efficiency.

By following these steps, you will be able to assess the performance of the DLD chip in terms of cell recovery and purity, ensuring that the separation efficiency is accurately measured and documented.

S5. Fluorescence identification

Reagents

Triton-X100 (Beyotime)，Mouse anti-chicken Pax7 (Santa Cruz Biotechnology)，

FITC-labeled goat anti-mouse IgG (H+L) (Beyotime)，DAPI (Beyotime)，

Experimental Procedure:

**Centrifugation:** Centrifuge the liquids separated by different channels of the microfluidic chip at 1500 rpm for 5 minutes. Discard the supernatant cell culture medium.

**Fixation:** Wash the cells twice with PBS.

Fix the cells with 4% paraformaldehyde in the dark for 10 minutes.

Wash the cells three times with PBST.

**Permeabilization:** Treat the cells with 0.5% Triton-X100 for 2 minutes for permeabilization.

Wash the cells three times with PBST.

**Blocking:** Block the cells with 1% BSA for 30 minutes.

Wash the cells three times with PBST.

**Primary Antibody Incubation:** Add mouse anti-chicken Pax7, diluted to the working concentration (1:1000) with 1% BSA.

Incubate in a humid chamber at 37°C for 1 hour.

Wash the cells three times with PBST.

**Secondary Antibody Incubation:** Add FITC-labeled goat anti-mouse IgG (1:1000) diluted with 1% BSA. Incubate in a humid chamber at 37°C in the dark for 1 hour.

Wash the cells three times with PBST.

**Nuclear Staining:** Discard the secondary antibody and wash the cells three times with PBST, 5 minutes each.

Stain the cells with DAPI (1:1000) in 1% BSA at room temperature in the dark for 10 minutes.

Wash the cells three times with PBST, 5 minutes each.

**Mounting and Detection:** Mount the cells with glycerol.

Capture images and count the cells under a fluorescence microscope.

Observe and record the number of various cells in each channel.

By following these steps, you can ensure accurate detection and analysis of cells separated by the microfluidic chip, observing the effectiveness of the separation process under a fluorescence microscope.
