# Supplementary material for: Rapid high-throughput isolation and purification of chicken myoblasts based on deterministic lateral displacement microfluidic chips
Source: PLoS One. 2024 Dec 5;19(12):e0301309. doi: 10.1371/journal.pone.0301309 (PMC11620410; doi:10.1371/journal.pone.0301309)
Supplement: Attachment — Submitted filename: Response to Journal and Reviewers.docx [file pone.0301309.s002.docx]

Response

Journal requirements:

When submitting your revision, we need you to address these additional requirements.

1.Please ensure that your manuscript meets PLOS ONE's style requirements, including those for file naming. The PLOS ONE style templates can be found at

https://journals.plos.org/plosone/s/file?id=wjVg/PLOSOne_formatting_sample_main_body.pdf and

<https://journals.plos.org/plosone/s/file?id=ba62/PLOSOne_formatting_sample_title_authors_affiliations.pdf>"

R1: Thank you very much for your suggestions. We follow your link and modify the manuscript format.

2.Thank you for stating the following financial disclosure:

'This research is funded by the National Key R&D Program of China (Grant No.2021YFD1300100), the Key R&D projects in Hainan Province (ZDYF2023XDNY036, ZDYF2022SHFZ033 and ZDYF2022SHFZ301), the Project of Wenchang city Wenchang Chickens Research Institute (WWXM20230301), and the Animal Branch of the Germ plasm Bank of Wild Species, Chinese Academy of Sciences."

Please state what role the funders took in the study. If the funders had no role, please state: "The funders had no role in study design, data collection and analysis, decision to publish, or preparation of the manuscript."

If this statement is not correct you must amend it as needed.

Please include this amended Role of Funder statement in your cover letter; we will change the online submission form on your behalf.

R2: Thank you very much for your suggestions regarding this part of the manuscript. We add relevant statements, and see line 411-413.

3. We note that your Data Availability Statement is currently as follows: [All relevant data are within the manuscript and its Supporting Information files.]

Please confirm at this time whether or not your submission contains all raw data required to replicate the results of your study. Authors must share the “minimal data set” for their submission. PLOS defines the minimal data set to consist of the data required to replicate all study findings reported in the article, as well as related metadata and methods (https://journals.plos.org/plosone/s/data-availability#loc-minimal-data-set-definition).

For example, authors should submit the following data:

- The values behind the means, standard deviations and other measures reported;

- The values used to build graphs;

- The points extracted from images for analysis.

Authors do not need to submit their entire data set if only a portion of the data was used in the reported study.

If your submission does not contain these data, please either upload them as Supporting Information files or deposit them to a stable, public repository and provide us with the relevant URLs, DOIs, or accession numbers. For a list of recommended repositories, please see https://journals.plos.org/plosone/s/recommended-repositories.

If there are ethical or legal restrictions on sharing a de-identified data set, please explain them in detail (e.g., data contain potentially sensitive information, data are owned by a third-party organization, etc.) and who has imposed them (e.g., an ethics committee). Please also provide contact information for a data access committee, ethics committee, or other institutional body to which data requests may be sent. If data are owned by a third party, please indicate how others may request data access.

R3: Thank you very much for your suggestions regarding this part of the manuscript. The required experimental data are packaged into the Excel file "experimental data".

4.Your ethics statement should only appear in the Methods section of your manuscript. If your ethics statement is written in any section besides the Methods, please move it to the Methods section and delete it from any other section. Please ensure that your ethics statement is included in your manuscript, as the ethics statement entered into the online submission form will not be published alongside your manuscript.

[Note: HTML markup is below. Please do not edit.]

R4: Thank you very much for your suggestions regarding this part of the manuscript. As you mentioned, ethics statement is moved to "Materials and Methods" see lines 160-164.

5. Review Comments to the Author

Please use the space provided to explain your answers to the questions above. You may also include additional comments for the author, including concerns about dual publication, research ethics, or publication ethics. (Please upload your review as an attachment if it exceeds 20,000 characters)

Reviewer #1: 1. The introduction includes technical terms and concepts related to microfluidic technology and the isolation of myoblast cells. While it's important to include this detailed information, make sure to explain it clearly for readers who may not be familiar with the terms. Consider providing brief explanations or definitions for terms like "Deterministic Lateral Displacement" (DLD) and "Mesoderm-derived muscle progenitor cells" to enhance understanding.

R1: Thank you very much for your suggestions regarding this part of the article. In the field of microfluidics, "Deterministic Lateral Displacement (DLD)" is a key technology that involves precise control of the lateral movement of fluids or particles at the micron or nanometer scale. see line 103-105.

Additionally, "mesoderm-derived muscle progenitor cells" refer to a group of cells that originate from the mesoderm during embryonic development and have the potential to differentiate into muscle cells. see line 83-91.

2. Check for grammar errors, awkward phrasing, and language clarity in the text. Ensure that each sentence is clear and concise, and that the overall language is suitable for a scientific audience.

R2: Thank you very much for your suggestions. We have made some revisions in the manuscript. For example, 132-134，225-226 line et al.

3. Detailed experimental protocols: Provide detailed protocols for each experimental procedure, including tissue sectioning, staining, cell suspension preparation, chip design, and DLD separation. This includes precise information on incubation times, temperatures, reagent concentrations, and specific equipment settings.

R3: Thank you very much for your suggestions. The detailed scheme of our experiment is put in the supplementary materials. Detailed protocol for each experimental procedure, includes tissue sections, staining, cell suspension preparation, microarray fabrication, deterministic lateral displacement (DLD) isolation, fluorescence identification.

Reviewer #2: 1. The abstract should clearly state the significance and novelty of the proposed microfluidic method for myoblast cell isolation and purification.

R1: Thank you very much for your suggestions for this part of the manuscript. The abstract emphasizes the importance and novelty of microfluidic cells in myoblast isolation and purification. See line 63-66.

2. Provide more detailed information on the design and fabrication process of the DLD chip, including specific dimensions and techniques used.

R2: Thank you very much for your suggestions. The detailed scheme of our experiment is put in the Supplementary materials, which provide a detailed scheme for each experimental procedure, including deterministic lateral displacement chip processing, manufacturing main methods and steps, and some size parameters of the chip, circular microcolumn diameter, circular microcolumn spacing, deflection angle, lateral spacing λ, longitudinal spacing.

3. Include statistical analysis and data validation in the results section to ensure the robustness of the findings.

R3: Thank you very much for your suggestions for this part of the manuscript. In the study of the microfluidic method for myoblast isolation and purification, the descriptive statistics of cell number, purity, and recovery were used to describe the basic features of the cell purification process. Three independent repeated experiments were performed simultaneously to verify the repeatability and consistency of the experimental results, and the statistical analysis and data validation are the key steps to ensure the robustness of the results. Please see the " experimental data " section.

4. Add a brief section addressing the limitations of the method and suggesting avenues for further optimization and improvement.

R4: Thank you very much for your suggestions for this part of the article. This method has some limitations, consider some improvement and optimization measures. As in the manuscript, the line 411-413.
