# Supplementary material for: Rapid high-throughput isolation and purification of chicken myoblasts based on deterministic lateral displacement microfluidic chips
Source: PLoS One. 2024 Dec 5;19(12):e0301309. doi: 10.1371/journal.pone.0301309 (PMC11620410; doi:10.1371/journal.pone.0301309)
Supplement: Attachment — Submitted filename: Response to Reviewers 0801.docx [file pone.0301309.s003.docx]

Responses to comments

Rapid High-Throughput Isolation and purification of Chicken Myoblasts Based on Deterministic Lateral Displacement Microfluidic Chips

PLOS ONE

Journal Requirements:

Please review your reference list to ensure that it is complete and correct. If you have cited papers that have been retracted, please include the rationale for doing so in the manuscript text, or remove these references and replace them with relevant current references. Any changes to the reference list should be mentioned in the rebuttal letter that accompanies your revised manuscript. If you need to cite a retracted article, indicate the article’s retracted status in the References list and also include a citation and full reference for the retraction notice.
R1: Thank you very much for your suggestions. The withdrawn references were removed and replaced with the relevant current references. Modified the references 488-494 line in the Manuscript.

6. Review Comments to the Author

Please use the space provided to explain your answers to the questions above. You may also include additional comments for the author, including concerns about dual publication, research ethics, or publication ethics. (Please upload your review as an attachment if it exceeds 20,000 characters)

Reviewer #1: Reviewer's Comments

Thank you for your trust. Let me review this draft. This article still has some problems. The following are my review comments.

1. The explanation of the Deterministic Lateral Displacement (DLD) technique is highly technical and might be difficult for some readers to follow. Consider simplifying the language or providing additional explanatory text. Additionally, the reference to Fig. 1 could be enhanced by ensuring that the figure itself is clear and well-labeled, making it easier for readers to understand the concept without needing extensive background knowledge. Including a brief description of the key components (e.g., laminar flows, particle trajectories) in simpler terms would also be beneficial.

R1: Thank you very much for your suggestions. The professional terms in the Manuscript are introduced to facilitate the readers to understand. Add explanations of laminar flows and particle trajectories in lines 126-129 and 137-138 of the Manuscript.

2.To improve readability and clarity, consider organizing the content into clearly labeled subsections with descriptive headings. For example, you can create distinct subsections for "Animal Study Protocols," "Tissue Sectioning and Staining," "Preparation of Cell Suspensions," "Chip Design and Processing," and "Purification of Myoblast Cells by Microfluidics." This will help readers navigate the methodology more easily.
R2: Thank you very much for your suggestions. Following your suggestion, Content is organized as clearly marked subsections with descriptive titles. Add animal study protocols and optimize the headings.

3. Ensure that all critical parameters for reproducibility are included, such as the specific temperatures for paraffin embedding and the precise composition of buffers and solutions used.
R3: Thank you very much for your suggestions. Following your suggestion, we have refined the details of the experiment .Include many experimental details in the Supplementary materials, such as paraffin processing.

4. Ensure that the descriptions of developmental stages and processes are clear and consistent throughout the text. For example, in the discussion of the transition from the proliferative stage to the fusion stage (E7–E15), clarify any ambiguous terms and consistently use scientific terminology to describe the stages of muscle development. This will help readers follow the progression of events without confusion.
R4: Thank you very much for your suggestions. The ambiguous terminology was clarified during the transition from the proliferation phase to the fusion phase (E7-E15). Modifications are shown in the Manuscript line 263-269.

5. Provide more detailed explanations for the figures referenced in the text. For example, when discussing Figures 4 and 5, include a brief description of the key observations from these figures and their significance to the study. This will help readers understand the context and relevance of the data presented in the figures without having to refer back and forth between the text and the figures.
R5: Thank you very much for your suggestions. For the readers to better understand the figures 4 and 5, to enrich the interpretation of the pictures. Add explanations in lines 280-281 and 283-284 of the Manuscript.
6. Ensure that the statistical analysis and data presentation are robust and transparent. For example, describe the statistical methods used to analyze the changes in myofiber diameter and the number of myofibers per unit area (Figures 4 and 5). Include any relevant statistical metrics, such as p-values or confidence intervals, to support the significance of the observed trends. Additionally, consider presenting the data in a more visually accessible format, such as using bar graphs or line charts with error bars, to clearly convey the trends and variability in the data.

R6: Thank you very much for your suggestions. Considering the completeness and rationality of the data, we improved on Figures 4 and 5. Add error bar to Figures 4 and 5 to enhance the reliability of the data.

[NOTE: If reviewer comments were submitted as an attachment file, they will be attached to this email and accessible via the submission site. Please log into your account, locate the manuscript record, and check for the action link "View Attachments". If this link does not appear, there are no attachment files.]

While revising your submission, please upload your figure files to the Preflight Analysis and Conversion Engine (PACE) digital diagnostic tool, <https://pacev2.apexcovantage.com/>. PACE helps ensure that figures meet PLOS requirements. To use PACE, you must first register as a user. Registration is free. Then, login and navigate to the UPLOAD tab, where you will find detailed instructions on how to use the tool. If you encounter any issues or have any questions when using PACE, please email PLOS at [figures@plos.org](mailto:figures@plos.org). Please note that Supporting Information files do not need this step.

R1: Thank you very much for your suggestions. There are some format problems in the previous pictures, so we revised those according to the website. The modified pictures will be added in the attachments.
